# Supplementary material for: Leptospirosis in pregnancy: A systematic review
Source: PLoS Negl Trop Dis. 2021 Sep 14;15(9):e0009747. doi: 10.1371/journal.pntd.0009747 (PMC8462732; doi:10.1371/journal.pntd.0009747)
Supplement: S1 Text — PROSPERO: CRD42020151501. https://www.crd.york.ac.uk/prospero/display_record.php?RecordID=151501. (PDF) [file pntd.0009747.s001.pdf]

Citation

Sujitha Selvarajah, Manisha Nair, Shaolu Ran, Nia Wyn Roberts. Leptospirosis in pregnancy: a systematic review. PROSPERO 2020 CRD42020151501 Available from:
https://www.crd.york.ac.uk/prospero/display_record.php?ID=CRD42020151501

Review question

Research questions:

1. What is the incidence of leptospirosis in pregnant women, globally?

2. What are the maternal and fetal/ infant outcomes of leptospirosis during pregnancy?

3. What is the evidence related to efficacy and safety of drugs for treating leptospirosis in pregnant women?

Searches

Electronic databases for published literature: MEDLINE, Embase, CINAHL (Cumulative Index to Nursing and Allied Health Literature), Global Health (OvidSP), Web of Science and Cochrane Central Register of Controlled Trials (CENTRAL)

Unpublished/ grey literature: The grey literature searches will be restricted to dissertations, reports and conference proceedings using recognised database ‘Proquest Dissertations & Theses’. In addition, ongoing unpublished trials will be identified by searching the trials registers such as www.ClinicalTrials.gov and www.who.int/trialsearch/

Hand searching of the reference list of included studies and key journals in the fields of obstetrics and infectious diseases.

Contact study authors for full report and/ or data if required

Time frame: From the inception of the databases until 28/02/20

Types of study to be included

Types of studies to be included: All study designs except ecological studies, expert opinion, correspondence and those describing mainly the pathology and pathogenesis of the infections. Also excluding case reports/series, correspondence. Also excluding papers focusing on preventative measures eg. vaccinations or travel advice for pregnant women as these do not answer the research questions.

Excluding literature reviews, but searching reference lists for any relevant articles.

Condition or domain being studied

Leptospirosis: Leptospirosis is a bacterial disease that affects humans and animals. It is caused by bacteria of the genus Leptospira. In humans, it can cause a wide range of symptoms, but some infected people may not have any symptoms. If untreated, it can lead to kidney damage, meningitis, liver failure, respiratory distress, and even death (CDC).

Participants/population

Types of participants

Inclusion criteria:

· Pregnant women diagnosed with leptospirosis

Exclusion criterion:

· Non-pregnant population diagnosed with leptospirosis

· Unconfirmed diagnosis of Leptospirosis

Setting: All countries.

Intervention(s), exposure(s)

Exposures: Leptospirosis diagnosed at any point during pregnancy.

Interventions: Medication options for Leptospirosis in pregnancy

Comparator(s)/control

For question 1, no comparator group

For question 2, pregnant women without leptospirosis

For question 3, placebo/standard treatment

Context

Setting: Global

Main outcome(s)

1. Rates of leptospirosis in pregnant women, globally.

2. Proportions of maternal and fetal/ infant complications (including mortality) associated with leptospirosis during pregnancy.

3. Risk ratios to compare the efficacy and safety of different drugs used for the treatment of leptospirosis during pregnancy.

Measures of effect

Research Question 1 - effect measures: Rates of infection in pregnant women and 95% Confidence Interval

Research Question 2 and 3 - effect measures: Odds/Risk Ratios/Proportions

Additional outcome(s)

None

Measures of effect

None

Data extraction (selection and coding)

SS and NR will independently carry out the searches in electronic databases. Bibliographic database Endnote, will be used to manage references, identify duplicates and share references among the reviewers. The screening will be carried out independently by two reviewers SS and SR in two stages – (i) screening of titles and abstracts based on the pre-specified inclusion and exclusion criteria; (ii) screening of full-texts of the papers included during stage-1. Any disagreements on inclusion of studies will be discussed and, where possible, resolved by consensus after referring to the review protocol; a third reviewer MN will be consulted if unresolved. A record of decisions made for each article will be maintained.

The search strategy will be piloted by applying this inclusion and exclusion criteria to a sample of papers to check its reliability in correctly classifying the studies:
Include:

· Pregnant women diagnosed with leptospirosis

Exclude:

· Non-pregnant population diagnosed with leptospirosis

· Unconfirmed diagnosis of leptospirosis

· Animal studies

· Correspondence

· Ecological studies

· Those describing mainly the pathology and pathogenesis of the infections

We were initially planning on exclude case reports and series, but due to small number of eligible articles, we included case reports/series.

A PRISMA flow chart will be used to summarise the selection process.

We developed a data extraction form that will be used to record the required information from the included full-texts. The form was pilot tested in 5% of the included studies and refined further. SS and SR will independently extract data and enter information on an Excel workbook.

The following information will be extracted from each included study:

· Sample size

· Serological markers eg. Leptospira antibodies, LFTs, Haemoglobin

· Exposures/Risk Factors

· Location

·Population

· Maternal outcomes eg. Fever, jaundice, deranged LFTs, malaise, death, postpartum haemorrhage, pregnancy-induced-hypertension, pre-eclampsia, eclampsia

· Neonatal outcomes eg, gestational age, delivery type, APGAR scores, birth weight, congenital infection, abortion

· Treatment used – effective/safe in pregnant women?

Risk of bias (quality) assessment

National Heart, Lung and Blood Institute's 'Study Quality Assessment Tools' for Observational Cohort and Cross-Sectional studies.

https://www.nhlbi.nih.gov/health-topics/study-quality-assessment-tools

Strategy for data synthesis

We will undertake a formal narrative synthesis to answer each research question. We will provide a description of the included studies that will include a description of the study population, country/ region,

sample size, incidence rates estimated for leptospirosis in pregnancy (numerator and denominator), maternal and child outcomes for women diagnosed with leptospirosis in pregnancy, and treatment used and its effectiveness. We will analyse the case reports, which do not have a comparator group, separately and present the results in a table. We will describe the quality of the included studies and present this information using a colour-coded chart. We will present a discussion of the strengths and limitations of the review including quality and publication bias.

Quantitative analysis: We plan to conduct a meta-analysis using random-effects model to estimate the pooled incidence rate (with 95% Confidence Interval (CI)) of leptospirosis in pregnancy. We will use random-effects model/ Peto method to calculate pooled risk ratios/ odds ratios (with 95% CI) to compare maternal and child outcomes in women diagnosed with leptospirosis during pregnancy compared with healthy controls, and effectiveness of drugs (antibiotics, mainly). The results will be presented in forest plots. We will report the I^2 (I^2) test statistic with 95% CI and interpret the variability in effect estimates that could be attributed to heterogeneity. Meta-analysis will be conducted using Stata SE, v15.

Criteria for data synthesis: A meta-analysis will be conducted if we have two or more studies that can be included in the analysis.

Analysing publication bias: We will use a standard funnel plot to analyse publication bias if five or more studies are included, as per Cochrane recommendation.

Analysis of subgroups or subsets

We will conduct subgroup analysis to examine the regional variation in rates of leptospirosis in pregnancy. We will investigate whether the maternal and fetal/ infant outcomes, and clinical management vary by gestational age (first trimester, second trimester or third trimester) at onset of the disease.

Contact details for further information

Sujitha Selvarajah
sujitha.selvarajah@npeu.ox.ac.uk

Organisational affiliation of the review

National Perinatal Epidemiology Unit (NPEU), Nuffield Department of Population Health, University of Oxford
https://www.npeu.ox.ac.uk/about

Review team members and their organisational affiliations

Dr Sujitha Selvarajah. National Perinatal Epidemiology Unit (NPEU), Nuffield Department of Population Health University of Oxford,
Dr Manisha Nair. National Perinatal Epidemiology Unit (NPEU), Nuffield Department of Population Health University of Oxford,
Dr Shaolu Ran. King's College London NHS Foundation Trust
Ms Nia Wyn Roberts. Bodleian Health Care Libraries, University of Oxford

Type and method of review

Meta-analysis, Systematic review

Anticipated or actual start date

18 November 2019

Anticipated completion date

14 March 2020

Funding sources/sponsors

The systematic review is funded by a Medical Research Council (GCRF) Career Development Award to Manisha Nair (Grant Ref: MR/P022030/1)

Conflicts of interest

Language

English

Country

England

Stage of review
Review Ongoing

Subject index terms status
Subject indexing assigned by CRD

Subject index terms
Female; Humans; Leptospirosis; Pregnancy

Date of registration in PROSPERO
11 March 2020

Date of first submission
02 February 2020

Stage of review at time of this submission

Stage	Started	Completed
Preliminary searches	Yes	No
Piloting of the study selection process	Yes	No
Formal screening of search results against eligibility criteria	Yes	No
Data extraction	No	No
Risk of bias (quality) assessment	No	No
Data analysis	No	No

The record owner confirms that the information they have supplied for this submission is accurate and complete and they understand that deliberate provision of inaccurate information or omission of data may be construed as scientific misconduct.
The record owner confirms that they will update the status of the review when it is completed and will add publication details in due course.

Versions
11 March 2020
