# Supplementary material for: Leptospirosis in pregnancy: A systematic review
Source: PLoS Negl Trop Dis. 2021 Sep 14;15(9):e0009747. doi: 10.1371/journal.pntd.0009747 (PMC8462732; doi:10.1371/journal.pntd.0009747)
Supplement: S2 Text — (DOCX) [file pntd.0009747.s002.docx]

# S2 Text. Search Strategy

**PUBMED**

1. “Leptospirosis”

2. "Weil’s Disease”

3. "Canicola Fever”

4. "Field Fever”

5. "Mud Fever”

6. “Spirochetosis”

7. “Swineherd's Disease”

8. “Rat Fever”

9. “Pretibial Fever”

10. “Rat Catcher’s Yellows”

11. “Canefield Fever”

12. “Nanukayami”

13. “Fort Bragg Fever”

14. “Seven Day Fever”

15. 1 OR 2 OR 3 OR 4 OR 5 OR 6 OR 7 OR 8 OR 9 OR 10 OR 11 OR 12 OR 13 OR 14

16. (animals [mh] NOT humans [mh])

17. 15 NOT 16

18. comment[pt] or editorial[pt] or letter[pt] or meta analysis[pt] or news[pt] or newspaper article[pt] or "review"[pt] or "systematic review"[pt]

19. 17 NOT 18

20. Pregnan*

21. prenat*

22. pre-nat*

23. antenat*

24. ante-nat

25. matern*

26. Partur*

27. gravid*

28. prepart*

29. peripart*

30. pre-part*

31. peri-part*

32. ante-part*

33. antepart*

34. intrapart*

35. intra-part*

36. 20 OR 21 OR 22 OR 23 OR 24 OR 25 OR 26 OR 27 OR 28 OR 29 OR 30 OR 31 OR 32 OR 33 OR 34 OR 35

37. 19 AND 36

**EMBASE**

1. Leptospirosis.af

2. Weil’s Disease.af.

3. Canicola Fever.af.

4. Field Fever.af

5. Mud Fever.af.

6. “Spirochet*”.af.

7. Swineherds Disease.af.

8. Rat Fever.af.

9. Pretibial Fever.af.

10. Fort Bragg Fever.af.

11. Seven Day Fever.af.

12. Canefield Fever.af.

13. Nanukayami.af

1. 1 or 2 or 3 or 4 or 5 or 6 or 7 or 8 or 9 or 10 or 11 OR 12 OR 13

2. (exp animals/ or nonhuman/) not human/

3. 12 NOT 13

4. Limit 16 to meta analysis

5. Limit 16 to systematic review

6. Limit 16 to editorial

7. Limit 16 to letter

8. Limit 16 to “review”

9. 17 OR 18 OR 19 OR 20 OR 21

10. 16 NOT 22

11. “Pregnan*”.af.

12. “prenat*”.af

13. “pre-nat*”.af

14. “antenat*”.af

15. “ante-nat*”.af

16. “matern*”.af

17. “Partur*”.af

18. “gravid*”.af

19. “prepart*”.af

20. “pre-part*”.af

21. “peripart*”.af

22. “peri-part*”.af

23. “ante-part*”.af

24. “antepart*”.af

25. “intrapart*”.af

26. “intra-part*”.af

27. 22 OR 23 OR 24 OR 25 OR 26 OR 27 OR 28 OR 29 OR 30 OR 31 OR 32 OR 33 OR 34 OR 35 OR 36 OR 37

28. 23 AND 40

Rat Catcher’s Yellows – unable to map term to subject heading

**CINAHL**

**S1: TX All Text:** ( Leptospirosis OR "Weil’s Disease" OR "Canicola Fever" OR "Field Fever" OR "Mud Fever" OR Spirochetosis OR "Swineherd's Disease" OR "Rat Fever" OR "Pretibial Fever" OR Nanukayami OR "Fort Bragg Fever" OR "Seven Day Fever" OR "Rat Catcher’s Yellows" OR "Canefield Fever"

AND

Tx All Text: ( "Pregnan*" OR "pre-nat*" OR "prenat*" OR "antenat*" OR "ante-nat*" OR "matern*" OR "partur*" OR "gravid*" OR "pre-part*" OR "prepart*" OR "peri-part*" OR "peripart*" OR "intrapart*" OR "intra-part*" OR "ante-part*" OR "antepart*" )

**S2: Limit S1 to Publication Type:** Commentary, Editorial, Letter, Meta Analysis, Meta Synthesis, Review, Systematic Review

**S3: S1 NOT S2**

**Web of Science**

**ALL FIELDS:** (Leptospirosis OR "Weils Disease" OR "Canicola Fever" OR "Field Fever" OR "Mud Fever" OR Spirochetosis OR "Swineherd's Disease" OR "Rat Fever" OR "Pretibial Fever" OR Nanukayami OR "Fort Bragg Fever" OR "Seven Day Fever" OR "Rat Catchers Yellows" OR "Canefield Fever") *AND* **ALL FIELDS:** (( "Pregnan*" OR "pre-nat*" OR "prenat*" OR "antenat*" OR "ante-nat*" OR "matern*" OR "partur*" OR "gravid*" OR "pre-part*" OR "prepart*" OR "peri-part*" OR "peripart*" OR "intrapart*" OR "intra-part*" OR "ante-part*" OR "antepart*" )

Excluding:

Review

Editorial Material

Letter

**Global Health Library**

1. Leptospirosis.af

2. Weils Disease.af.

3. Canicola Fever.af.

4. Field Fever.af

5. Mud Fever.af.

6. “Spirochet*”.af.

7. Swineherds Disease.af.

8. Rat Fever.af.

9. Pretibial Fever.af.

10. Fort Bragg Fever.af.

11. Seven Day Fever.af.

12. Canefield Fever.af.

13. Nanukayami.af

14. 1 or 2 or 3 or 4 or 5 or 6 or 7 or 8 or 9 or 10 or 11 OR 12 OR 13

15. exp animals/ not humans.mp

16. 14 NOT 15

17. Limit 16 to editorial

18. Limit 16 to correspondence

19. 17 OR 18

20. 16 NOT 19

21. “Pregnan*”.af.

22. “prenat*”.af

23. “pre-nat*”.af

24. “antenat*”.af

25. “ante-nat*”.af

26. “matern*”.af

27. “Partur*”.af

28. “gravid*”.af

29. “prepart*”.af

30. “pre-part*”.af

31. “peripart*”.af

32. “peri-part*”.af

33. “ante-part*”.af

34. “antepart*”.af

35. “intrapart*”.af

36. “intra-part*”.af

37. 19 OR 20 OR 21 OR 22 OR 23 OR 24 OR 25 OR 26 OR 27 OR 28 OR 29 OR 30 OR 31 OR 32 OR 33 OR 34

**38.** 20 AND 37

**Proquests and Dissertations & Theses Global 7**

“Leptospirosis” OR “Weil’s Disease” OR “Canicola Fever” OR “Field Fever” OR “Mud Fever” OR “Spirochetosis” OR “Swineherd's Disease” OR “Rat Fever” OR “Pretibial Fever” OR “Nanukayami” OR “Fort Bragg Fever” OR “Seven Day Fever” OR “Rat Catcher’s Yellows” OR “Canefield Fever”

**Anywhere but full text**

**AND**

Pregnan* OR pre-nat* OR prenat* OR antenat* OR ante-nat* OR matern* OR partur* OR gravid* OR pre-part* OR prepart* OR peri-part* OR peripart* OR intrapart* OR intra-part* OR ante-part* OR antepart*

**(anywhere)**

**WHO ICTRP 29**

“Leptospirosis”

<https://apps.who.int/trialsearch/>

**ClinicalTrials.gov 11**

“Leptospirosis”

<https://clinicaltrials.gov/ct2/results/details?cond=Leptospirosis>

Pregnan* OR prenat* OR pre-nat* OR antenat* OR ante-nat OR matern* OR Partur* OR gravid* OR prepart* OR peripart*OR pre-part* OR peri-part* OR ante-part* OR antepart* OR intrapart* OR intra-part*

Leptospirosis OR "Weils Disease" OR "Canicola Fever" OR "Field Fever" OR "Mud Fever" OR “Spirochet*” OR "Swineherds Disease" OR "Rat Fever" OR "Pretibial Fever" OR "Fort Bragg Fever" OR "Seven Day Fever" OR "Canefield Fever" OR "Nanukayami"
