# Supplementary material for: Leptospirosis in pregnancy: A systematic review
Source: PLoS Negl Trop Dis. 2021 Sep 14;15(9):e0009747. doi: 10.1371/journal.pntd.0009747 (PMC8462732; doi:10.1371/journal.pntd.0009747)
Supplement: S3 Text — (DOCX) [file pntd.0009747.s003.docx]

# S3 Text. Coding for Data Extraction from Case Reports and Series

**Leptospirosis exposure in history**

1= exposure specified

0 = nothing in history that is relevant/no exposure identified from history

NA = not available (i.e. poor quality papers with no history mentioned)

**Comorbidities**

1 = healthy with no comorbidities

0 = comorbidities present

NA = not available

**Abdominal pain**

1 = abdominal pain specified

0 = ‘rest of examination normal’/no abdominal or bodily pain

NA = not available

**Nausea or Vomiting**

1 = nausea or vomiting specified

0 = no other features/no nausea or vomiting

NA = not available

**Clinical jaundice**

1 = jaundice specified

0 = jaundice not mentioned/mentioned as negative symptom

NA = not available ie.no mention of examination

**Fever**

1 = febrile temperature >37.5C

0 = afebrile/normal temperature/all observations normal

NA = not available

**Malaise**

1 = malaise/tiredness specified

0 = ‘no other symptoms’ and no mention of malaise/tiredness

NA = not available

**Myalgia**

1 = aches/pains/myalgia specified

0 = ’no other symptoms’/pain mentioned as negative symptom

NA = not available

**Peripheral Oedema**

1 = oedema specified

0 = ‘rest of examination normal’ and no mention of oedema

NA = not available

**Headache**

1 = headache specified

0 = ‘rest of examination normal’ and no mention of headache

NA = not available

**Visual disturbance**

1 = visual disturbance specified

0 = says no visual disturbances/examination normal/neuro examination normal

NA = not available

**PV bleeding**

1= PV bleeding specified

0 = ‘rest of examination normal’/PV bleeding mentioned as negative symptom

NA = not available

**Conjunctival Suffusion**

1 = Conjunctival suffusion present or mention of red eyes

0 = No conjunctival suffusion stated/mention eye examination or some eye findings but no mention of conjunctival suffusion or state normal examination findings

NA = Cannot determine from information if there was conjunctival suffusion or eyes were examined

**Hypotension**

Defined as - SBP < 90 or MAP <70 or DBP <40 as per RCOG guidelines[[1]](https://paperpile.com/c/AOKnwE/T9z92)

1 = BP specified and hypotensive

O = normal BP specified/’rest of examination normal’

NA = blood pressure not available

**Hypertension**

Defined as - SBP> 140 or DBP > 90 as per RCOG guidelines[[1,2]](https://paperpile.com/c/AOKnwE/T9z92+qX9ZU)

1 = Author stated as hypertensive/ BP specified and hypertensive as per above threshold

0 = normal BP specified/’rest of examination normal’

NA = blood pressure not available

**Tachycardia**

Defined as - HR >100 as per RCOG guidelines[[1]](https://paperpile.com/c/AOKnwE/T9z92)

1 = HR specified and tachycardic

O = normal HR specified/’rest of examination normal’

NA = HR not available

**Oliguria**

- Defined as urine output <500mls/24hrs as per RCOG guidelines

1 = reduced urine output specified

0 = normal renal function or urine output specified

NA = urine output not available

**GCS reduced**

1 = confused/comatosed/GCS reduced specified

0 = alert and orientated at all times

NA = mental state not available

**Respiratory changes**

1 = symptoms/signs/imaging of respiratory changes specified

0 = ‘rest of examination normal’/respiratory changes mentioned as negative symptom

NA = respiratory changes not available

**Cardiac changes**

1 = symptoms/signs/imaging of cardiac changes specified

0 = ‘rest of examination normal’/cardiac changes mentioned as negative symptom

NA = cardiac changes not available

**Biochemical values**

1 = value stated as abnormal/lies outside of normal range

0 = value stated as normal/lies within normal range

NA = value not available

- Hyperbilirubinaemia = stated by author or clinically jaundiced or bilirubin level >0.8 mg/dL or >14 micromol/L[[3]](https://paperpile.com/c/AOKnwE/uARNw)
- Transaminitis = as stated by author or elevated transaminases/AST/ALT >40 IU/L [[4]](https://paperpile.com/c/AOKnwE/5LhKU)
- Coagulopathy = as stated by author or stated by author to have deranged clotting bloods (ie. prolonged APTT, deranged INR) or including a thrombocytopenia (defined below)
- Renal dysfunction = as stated by author or stated as significantly reduced urine output or Creatinine >97umol/L [[5,6]](https://paperpile.com/c/AOKnwE/gLuoW+71kxG) or doubling of creatinine in absence of renal disease [[7]](https://paperpile.com/c/AOKnwE/S1ubl)
- Anaemia defined as: <110g/L as per WHO[[8]](https://paperpile.com/c/AOKnwE/FBeWX)
- Leucocytosis = stated by author or WBC >12 x 10^9/L as per RCOG guidelines [[7]](https://paperpile.com/c/AOKnwE/S1ubl)
- Neutrophilia = as stated by author
- Thrombocytopaenia = as stated by author or platelet count <100 x 10^9 (RCOG)[[7]](https://paperpile.com/c/AOKnwE/S1ubl)

**Proteinuria**

1 = proteinuria specified or protein/creatinine ratio >0.3 or dipstick reading more 1+ [[7]](https://paperpile.com/c/AOKnwE/S1ubl)

0 = ‘normal urine dip’/proteinuria specified as negative sign

NA = not available

**IgM Serology**

1 = serological confirmation of IgM either as ELISA or specified IgM otherwiseNA = serological confirmation but not derivable if IgM present or tested for

**Isolated leptospira in placenta**

1 = placenta tested for leptospira and identified

0 = placenta tested for leptospira and not identified

NA = not derivable if leptospira in placenta/not tested for

**Urine Culture**

1 = urine cultured for leptospira and identified

0 = urine cultured for leptospira and not identified

NA = not derivable if leptospira present in urine culture/urine not cultured or not mentioned/urine

cultured but no mention of specific medium for leptospira

**Blood Culture**

1 = blood cultures sterile

0 = blood cultured for leptospira and identified

NA = not derivable if leptospira present in blood culture/blood not cultured or not mentioned

**Maternal ICU**

1 = admission to ICU or intubation specified

0 = no mention of admission to ICU or intubation/mentioned as no ICU admission

**Maternal Death**

1 = maternal death specified

0 = no maternal death

**Birth outcome**

1 = No Live birth

0 = Live Birth specified

**Fetal/neonatal outcome**

1= Fetal Death/IUFD (Intrauterine Fetal Death/Stillborn/neonatal death/miscarriage

0 = Live birth and lives through neonatal period

**Swansea Criteria for AFLP** [**[3]**](https://paperpile.com/c/AOKnwE/uARNw)

- Defined as 6 or more of:
  - Vomiting
  - Abdominal pain
  - Polydipsia/polyuria
  - Encephalopathy
  - Elevated bilirubin (>0.8 mg/dL or >14 micromol/L)
  - Hypoglycemia (<72 mg/dL or >4 mmol/L)
  - Leucocytosis (>11 x 10^9/L)
  - Elevated transaminases (AST or ALT) (>42 IU/L)
  - Elevated ammonia (>47 micromol/L)
  - Elevated urate (5.7 mg/dL or >340 micromol/L)
  - Acute kidney injury, or creatinine 1.7 mg/dL or >150 micromol/L
  - Coagulopathy or prothrombin time >14 seconds
  - Ascites or bright liver on ultrasound scan
  - Microvesicular steatosis on liver biopsy

NA = score not derivable

**AFLP differential diagnosis**

1 = AFLP as differential diagnosis specified and/or Swansea criteria met

0 = AFLP as differential diagnosis not specified/Swansea criteria not met

NA = information not available

**HELLP differential diagnosis**

- Defined as AST/ALT >40 IU/L and platelets <150 x 10^9/L as per Mississippi/Tennessee criteria[[4]](https://paperpile.com/c/AOKnwE/5LhKU)

1 = HELLP as differential diagnosis specified/HELLP criteria met

0 = HELLP as differential diagnosis not specified/HELLP criteria not met

NA = information not derivable

**PET** [**[5,6]**](https://paperpile.com/c/AOKnwE/gLuoW+71kxG)

- Defined as hypertension and one or more of the following complications after the 20th week of pregnancy:
  - proteinuria
  - Platelets < 100 x 10^9/L
  - Impaired liver function

Renal dysfunction - Creatinine >97umol/L or doubling of creatinine in the absence of renal disease, or urine output reduced to less than 500mls in 24hrs

- - Pulmonary oedema
  - Cerebral or visual disturbances

1 = PET as differential diagnosis specified/PET criteria met

0 = PET as differential diagnosis not specified/PET criteria not met

## NA = information not derivable

## References:

1. [Bacterial Sepsis in Pregnancy Green–top Guideline No. 64a, RCOG. Apr 2012 [cited 16 Apr 2021]. Available:](http://paperpile.com/b/AOKnwE/T9z92) <https://www.rcog.org.uk/globalassets/documents/guidelines/gtg_64a.pdf>

2. [Visual summaries | Hypertension in pregnancy: diagnosis and management | Guidance | NICE. [cited 16 Apr 2021]. Available:](http://paperpile.com/b/AOKnwE/qX9ZU) <https://www.nice.org.uk/guidance/ng133/resources/visual-summaries-8720711389>

3. [Westbrook RH, Dusheiko G, Williamson C. Pregnancy and liver disease. J Hepatol. 2016;64: 933–945. doi:](http://paperpile.com/b/AOKnwE/uARNw)[10.1016/j.jhep.2015.11.030](http://dx.doi.org/10.1016/j.jhep.2015.11.030)

4. [Haram K, Svendsen E, Abildgaard U. The HELLP syndrome: clinical issues and management. A Review. BMC Pregnancy Childbirth. 2009;9: 8. doi:](http://paperpile.com/b/AOKnwE/5LhKU)[10.1186/1471-2393-9-8](http://dx.doi.org/10.1186/1471-2393-9-8)

5. [Severe Pre-eclampsia/Eclampsia, Management (Green-top Guideline No. 10A). [cited 21 Apr 2021]. Available:](http://paperpile.com/b/AOKnwE/gLuoW) <https://www.rcog.org.uk/en/guidelines-research-services/guidelines/gtg10a/>

6. [Overview | Hypertension in pregnancy: diagnosis and management | Guidance | NICE. [cited 21 Apr 2021]. Available:](http://paperpile.com/b/AOKnwE/71kxG) <https://www.nice.org.uk/guidance/ng133>

7. Bacterial Sepsis in Pregnancy | RCOG Green-top Guideline No. 64a. [[cited 21 Apr 2021]. Available:](http://paperpile.com/b/AOKnwE/S1ubl) <https://www.rcog.org.uk/globalassets/documents/guidelines/gtg_64a.pdf>

8. [WHO recommendations on antenatal care for a positive pregnancy experience: evidence base*. [cited 21 Apr 2021]. Available:](http://paperpile.com/b/AOKnwE/FBeWX) <https://apps.who.int/iris/bitstream/handle/10665/250796/9789241549912-websupplement-eng.pdf?sequence=8>
