# Supplementary material for: Leptospirosis in pregnancy: A systematic review
Source: PLoS Negl Trop Dis. 2021 Sep 14;15(9):e0009747. doi: 10.1371/journal.pntd.0009747 (PMC8462732; doi:10.1371/journal.pntd.0009747)
Supplement: S1 Table — (DOCX) [file pntd.0009747.s004.docx]

# S1 Table. [Quality assessment tool for case reports and case series](https://docs.google.com/document/d/1bgWEKFrXw8CwHdV_-bVBSsywwgw6HAC4CIxaVOXgW9U/edit#heading=h.jnnvgfk0quyt)

This is the criteria used to assess the quality of the case reports and series. It is a modified version of the National Heart, Lung and Blood Institute[[1]](https://paperpile.com/c/AOKnwE/4AxbB), with adaptations including criteria from a tool published in the British Medical Journal Evidence Based Medicine [[2]](https://paperpile.com/c/AOKnwE/89Kf0).

An explanation of how the tool was devised is demonstrated with colour coding:

Green text means it is from [nhlbi tool](https://www.nhlbi.nih.gov/health-topics/study-quality-assessment-tools) [[1]](https://paperpile.com/c/AOKnwE/4AxbB)

Black text is from the [BMJ paper tool](https://ebm.bmj.com/content/23/2/60#ref-11) [[2]](https://paperpile.com/c/AOKnwE/89Kf0)

Orange text is criteria that was from both tools

| If Case Report Use Criteria 1-6. If Case Series, use 1-10 |  |
| --- | --- |
| 1. Was the exposure adequately ascertained? Ie. Serological leptospirosis confirmation |  |
| 2. Was the outcome adequately ascertained? (if series, was it implemented consistently across all study participants)  Ie. LFTs/Hb/Abortion |  |
| 3. If applicable, was the intervention clearly described? Delivery/Abx |  |
| 4. Was follow-up long enough for outcomes to occur? Ie. For titres to come down/clinical picture to improve |  |
| 5. Were the results well described? |  |
| 6. Is the case(s) described with sufficient details to allow other investigators to replicate the research or to allow practitioners make inferences related to their own practice? (Clear decision making process) |  |
| 7. CS -If applicable, were statistical methods well-described? |  |
| 8. CS - If case series, was the study population clearly and fully described, including a case definition? |  |
| 9. CS - If case series, were the cases consecutive? |  |
| 10. CS - If case series, were the subjects comparable? |  |

## References:

1. [Study Quality Assessment Tools. [cited 16 Apr 2021]. Available:](http://paperpile.com/b/AOKnwE/4AxbB) <https://www.nhlbi.nih.gov/health-topics/study-quality-assessment-tools>
2. [Murad MH, Sultan S, Haffar S, Bazerbachi F. Methodological quality and synthesis of case series and case reports. BMJ Evid Based Med. 2018;23: 60–63. doi:](http://paperpile.com/b/AOKnwE/89Kf0)[10.1136/bmjebm-2017-110853](http://dx.doi.org/10.1136/bmjebm-2017-110853)
