# Supplementary material for: Leptospirosis in pregnancy: A systematic review
Source: PLoS Negl Trop Dis. 2021 Sep 14;15(9):e0009747. doi: 10.1371/journal.pntd.0009747 (PMC8462732; doi:10.1371/journal.pntd.0009747)
Supplement: S3 Table — (DOCX) [file pntd.0009747.s006.docx]

# S3 Table. [Summary of ongoing studies](https://docs.google.com/document/d/1bgWEKFrXw8CwHdV_-bVBSsywwgw6HAC4CIxaVOXgW9U/edit#heading=h.tnxnztvgrtqv)

| **Results summary of ongoing studies** | | | | | |
| --- | --- | --- | --- | --- | --- |
| **ID** | **Name** | **Summary** | **Region** | **Start Date** | **End Date** |
| **NCT04288674** | [Leptospirosis Registry Gathers Knowledge on Epidemiology, Clinical Course, Prognostic Factors and Molecular Characteristics for Invasive Leptospirosis Disease](https://clinicaltrials.gov/ct2/show/NCT04288674) | World-wide retrospective observational (case-control) study addressing the increasing incidence of leptospirosis outbreak and local changes in species distribution. The LeptoScope registry aims to improve knowledge on epidemiology, clinical course prognostic factors and molecular characteristics for invasive leptospirosis disease. Primary outcomes: 1. Global incidence 2. Global mortality | EUR | March 2020 | (estimated) December 2030 |
| **NCT04034550** | [Cohort of Hospitalized Patients Suspected of Leptospirosis](https://clinicaltrials.gov/ct2/show/NCT04034550) | Single-centre prospective interventional study assessing participating acute leptospirosis cases during one year follow up for epidemiological, clinical, bacteriological and immunological data through blood, urine sampling and blood banking. Primary outcome: 1. Disease severity (rate of death or severe organ insufficiency) | EUR | (estimated) September 2019 | (estimated) September 2025 |
| **NCT03912506** | [Severe Leptospirosis in Non-tropical Areas](https://clinicaltrials.gov/ct2/show/NCT03912506) | Multi-centre retrospective observational (cohort) study of patients admitted to ICU for leptospirosis aiming to identify the characteristics, treatments and prognostic factors associated with mortality of severe leptospirosis. Primary outcome: 1. Mortality at ICU discharge | EUR | May 2017 | September 2018 |
| **NCT01080989** | [The Sero-Prevalence and Genetic Study for the Infectious Diseases and Metabolic Syndrome in Solomon Islands](https://apps.who.int/trialsearch/Trial2.aspx?TrialID=NCT01080989) | Single centre non-blinded interventional study aiming to collect data from the Solomon Islands to better understand health needs, characteristics of disease and basic health information so that strategies and operation goals for further improvement will be more explicit for the centre’s medical teams.The project uses blood samples, stool samples and a health screening questionnaire to 1. Health screen 2. Clinical diagnosis and treatment. Primary outcome: 1. Health screening for the community | WPR | March 2009 | December 2011 |
