# Supplementary material for: Leptospirosis in pregnancy: A systematic review
Source: PLoS Negl Trop Dis. 2021 Sep 14;15(9):e0009747. doi: 10.1371/journal.pntd.0009747 (PMC8462732; doi:10.1371/journal.pntd.0009747)
Supplement: S4 Table — (DOCX) [file pntd.0009747.s007.docx]

# S4 Table. [Record of all included Case Reports and Series](https://docs.google.com/document/d/1bgWEKFrXw8CwHdV_-bVBSsywwgw6HAC4CIxaVOXgW9U/edit#heading=h.186ohlbfkvrl)

| Record of all included Case Reports and Series | | |
| --- | --- | --- |
| **CASE REPORTS** | | |
|  | References | Number of cases |
| CR1 | 1. Gaspari R, Annetta MG, Cavaliere F, Pallavicini F, Grillo R, Conti G, Antonelli M, Tafani C, Proietti R. Unusual presentation of leptospirosis in the late stage of pregnancy. Minerva Anestesiol. 2007 Jul-Aug;73(7-8):429-32. PMID: 17637589. | 1 |
| CR2 | 1. Tramoni G, Clément HJ, Lopez F, Viale JP. Une cause inhabituelle d'hémorragie de la délivrance: la leptospirose [An unusual case of post partum haemorrhage: leptospirosis infection]. Ann Fr Anesth Reanim. 2003 Apr;22(4):363-5. French. doi: 10.1016/s0750-7658(03)00061-3. PMID: 12818331. | 1 |
| CR3 | 1. Hicham S, Ihsane M, Abderahim el B, Brahim B, Labib S, Mustapha H, Mohamed K, Adil I, Abdelilah M, Nabil K. Multivisceral organ failure related to leptospirosis in pregnant patient. Indian J Crit Care Med. 2013 Jan;17(1):43-5. doi: 10.4103/0972-5229.112143. PMID: 23833476; PMCID: PMC3701397. | 1 |
| CR4 | 1. Shaked Y, Shpilberg O, Samra D, Samra Y. Leptospirosis in pregnancy and its effect on the fetus: case report and review. Clin Infect Dis. 1993 Aug;17(2):241-3. doi: 10.1093/clinids/17.2.241. PMID: 8399874. | 1 |
| CR5 | 1. Dadhwal V, Bahadur A, Deka D. Leptospirosis as a cause of fever in pregnancy. Int J Gynaecol Obstet. 2007 Dec;99(3):252-3. doi: 10.1016/j.ijgo.2007.05.036. Epub 2007 Sep 21. PMID: 17888438. | 1 |
| CR6 | 1. Gainder S, Singla R, Dhaliwal L, Suri V. Leptospirosis as a cause of intrauterine fetal demise: short report of rare presentation. Arch Gynecol Obstet. 2010 Jun;281(6):1061-3. doi: 10.1007/s00404-009-1266-y. Epub 2009 Oct 30. PMID: 20440597. | 1 |
| CR7 | 1. Coghlan JD, Bain AD. Leptospirosis in human pregnancy followed by death of the foetus. Br Med J. 1969 Jan 25;1(5638):228-30. doi: 10.1136/bmj.1.5638.228. PMID: 5762626; PMCID: PMC1982050. | 1 |
| CR8 | 1. Koe SL, Tan KT, Tan TC. Leptospirosis in pregnancy with pathological fetal cardiotocography changes. Singapore Med J. 2014 Feb;55(2):e20-4. doi: 10.11622/smedj.2013194. PMID: 24712035; PMCID: PMC4291937. | 1 |
| CR9 | 1. Rahimi R, Omar E, Tuan Soh TS, Mohd Nawi SFA, Md Noor S. Leptospirosis in pregnancy: A lesson in subtlety. Malays J Pathol. 2018 Aug;40(2):169-173. PMID: 30173235. | 1 |
| CR10 | 1. [N. Aker, Elizabeth B. James, A. M. Johnston & G. Pasvol (1996) Leptospirosis in pregnancy: An unusual and relatively unrecognised cause of intrauterine death in man, Journal of Obstetrics and Gynaecology, 16:3, 163-165, DOI: 10.3109/01443619609004093](https://doi.org/10.3109/01443619609004093) | 1 |
| CR11 | 1. Chedraui PA, San Miguel G. A case of leptospirosis and pregnancy. Arch Gynecol Obstet. 2003 Nov;269(1):53-4. doi: 10.1007/s00404-002-0415-3. Epub 2002 Oct 2. PMID: 14605821. | 1 |
| CR12 | 1. Suzuki K, Nakamura S, Watanabe H. A fatal case of Leptospira autumnalis infection in Lao PDR. Southeast Asian J Trop Med Public Health. 1997 Jun;28(2):436-7. PMID: 9444037. | 1 |
| CR13 | 1. Faine S, Adler B, Christopher W, Valentine R. Fatal congenital human leptospirosis. Zentralbl Bakteriol Mikrobiol Hyg A. 1984 Sep;257(4):548. doi: 10.1016/s0176-6724(84)80091-7. PMID: 6542285. | 1 |
| CR14 | 1. LINDSAY S, LUKE IW. Fatal leptospirosis (Weil's disease) in a newborn infant; case of intrauterine fetal infection with report of an autopsy. J Pediatr. 1949 Jan;34(1):90-4. doi: 10.1016/s0022-3476(49)80206-x. PMID: 18127164. | 1 |
| CR15 | 1. Rathnaweera, R.H.A.I., 2015. A death of a pregnant mother following Leptospirosis. Medico-Legal Journal of Sri Lanka, 1(3), pp.20–22. DOI:<http://doi.org/10.4038/mljsl.v1i3.7303> | 1 |
| CR16 | 1. Baytur YB, Lacin S, Koyuncu FM, Cabuk M, Cabuk M, Ceylan C, Kandiloglu AR. Weil's syndrome in pregnancy. Eur J Obstet Gynecol Reprod Biol. 2005 Mar 1;119(1):132-3. doi: 10.1016/j.ejogrb.2004.06.021. PMID: 15734102. | 1 |
| CR17 | 1. BLEIER W, LECHTKEN FJ. Menschliche Leptospira-canicola-Infektion als Ursache eines Spontanabortes [Leptospirosis canicola in man as a cause of spontaneous abortion]. Geburtshilfe Frauenheilkd. 1951 Jun;11(6):538-44. Undetermined Language. PMID: 14849774. | 1 |
| CR18 | 1. Gsell HO Jr, Olafsson A, Sonnabend W, Breer C, Bachmann C. Intrauterine Leptospirosis pomona. Erster berichtter Fall einer intrauterin übertragenen und geheilten Leptospirose [Intrauterine leptospirosis pomona. 1st reported case of an intrauterine transmitted and cured leptospirosis]. Dtsch Med Wochenschr. 1971 Jul 30;96(31):1263-8. German. doi: 10.1055/s-0028-1110120. PMID: 4934402. | 1 |
| CR19 | 1. Walker, J.F., Walsh, J., Cronin, C.J. et al. Acute renal failure in leptospirosis. I.J.M.S. 150, 187 (1981). https://doi.org/10.1007/BF02938231 | 1 |
| CR20 | 1. Colette, C. (1962). "Fatal leptospirosis during pregnancy with severe jaundice.]." Bull Fed Soc Gynaecol Obstetric Lang Fr 14: 437-440 | 1 |
| CR21 | 1. TONG, C., MATHUR, M.. Leptospirosis in Pregnancy: A Rare Condition Mimicking HELLP Syndrome. Journal of Medical Cases, North America, 9, jun. 2018. Available at: <https://www.journalmc.org/index.php/JMC/article/view/3073/2407>. Date accessed: 25 Mar. 2021. | 1 |
| **CASE SERIES** | | |
| CS1 | 1. Olivares Martin J., Gomez Andres A., Luna Morales A., et al. Leptospirosis icterohemorrágica. Presentación de dos casos, 1977, Revista Clinica Española, Toma 147, Núm 4 | 1 (1 case excluded as no leptospirosis in pregnancy,therefore quality assessed as a case report) |
| CS2 | 1. Carles G, Montoya E, Joly F, Peneau C. Leptospiroses et grossesse. Etude de 11 cas en Guyane Française [Leptospirosis and pregnancy. Eleven cases in French Guyana]. J Gynecol Obstet Biol Reprod (Paris). 1995;24(4):418-21. French. PMID: 7650320. | 11 |
| CS3 | 1. VECCHIETTI G. Su due casi di leptospirosi in gravidanza [Two cases of leptospirosis in pregnancy]. Minerva Ginecol. 1952 Jan;4(1):9-12. Undetermined Language. PMID: 14940828. | 2 |
