# Supplementary material for: Leptospirosis in pregnancy: A systematic review
Source: PLoS Negl Trop Dis. 2021 Sep 14;15(9):e0009747. doi: 10.1371/journal.pntd.0009747 (PMC8462732; doi:10.1371/journal.pntd.0009747)
Supplement: S5 Table — (DOCX) [file pntd.0009747.s008.docx]

# S5 Table. [Record of excluded articles](https://docs.google.com/document/d/1bgWEKFrXw8CwHdV_-bVBSsywwgw6HAC4CIxaVOXgW9U/edit?pli=1#heading=h.vasco6t8bzuk)

| Type of record | References | Reason for exclusion |
| --- | --- | --- |
| General Records | 1. Jayakumar M, Prabahar MR, Fernando EM, Manorajan R, Venkatraman R, Balaraman V. Epidemiologic trend changes in acute renal failure--a tertiary center experience from South India. Ren Fail. 2006;28(5):405-10. doi: 10.1080/08860220600689034. PMID: 16825090. | No leptospirosis in pregnancy |
|  | 1. Leptospirosis following heavy rains in 2017 in Mumbai: Report of large-scale community chemoprophylaxis. Supe Avinash, Khetarpal Mini, Naik Shantaram, Keskar Padmaja. Year : 2018 \| Volume: 31 \| Issue Number: 1 \| Page: 19-21 | No leptospirosis in pregnancy |
|  | 1. Centers for Disease Control and Prevention (CDC). Brief report: Leptospirosis after flooding of a university campus--Hawaii, 2004. MMWR Morb Mortal Wkly Rep. 2006 Feb 10;55(5):125-7. PMID: 16467778. | No leptospirosis in pregnancy |
|  | 1. Fiecek B, Lewandowska G, Rogulska U, Chmielewski T. Leptospirosis in Poland in the years 2014-2017 – characteristics of infections and epidemiological surveillance data. Przegl Epidemiol. 2018;72(3):303-312. doi: 10.32394/pe.72.3.7. PMID: 30394053. | No leptospirosis in pregnancy |
|  | 1. Murphy JF, O'Driscoll K. Therapeutic abortion: the medical argument. Ir Med J. 1982 Aug;75(8):304-6. PMID: 7129852. | No leptospirosis in pregnancy |
|  | 1. Hudemann H, Maas G. Epidemiologische Untersuchungen auf Leptospirose und Listeriose in einem landwirtschaftlich strukturierten Kreis [Epidemiologic studies of leptospirosis and listeriosis in an agricultural region]. Dtsch Gesundheitsw. 1970 Feb 27;25(8):362-5. German. PMID: 5535633. | No leptospirosis in pregnancy |
|  | 1. Karnad DR, Richards GA, Silva GS, Amin P; Council of the World Federation of Societies of Intensive and Critical Care Medicine. Tropical diseases in the ICU: A syndromic approach to diagnosis and treatment. J Crit Care. 2018 Aug;46:119-126. doi: 10.1016/j.jcrc.2018.03.025. Epub 2018 Mar 27. PMID: 29625787. | No leptospirosis in pregnancy |
|  | de Silva K, Jayaweera BA. Leptospiral problems in pregnancy. Ceylon Med J. 1970 Jun;15(2):96-103. PMID: 5494247. | No Full Text Available |
|  | Evaluation of leptospirosis as a cause of fulminant hepatic failure, S, Suryanarayana B. Ann Arbor, All India Institute of Medical Sciences, New Delhi (India)2004 | No Full Text Available |
|  | Tokarevich KN, Popova EM, Pupkevich-Diamant IaS. K voprosu o likhoradochnoĭ reaktsii organizma pri leptospiroznoĭ infektsii. 1. Likhoradochnaia reaktsiia pri ikterogemorragicheskom leptospiroze [On the problem of fever reaction of the organism in leptospirosis infection. 1. Fever reaction in icterohemorrhagic leptospirosis]. Tr Leningr Nauchnoissled Inst Epidemiol Mikrobiol. 1966;29:44-63. Russian. PMID: 6015037. | No Full Text Available |
|  | DELTHIL P. Embryopathie à la suite d'un ictère infectieux fébrile à rechute [Embryopathy following Weil's disease during pregnancy]. Arch Fr Pediatr. 1951;8(8):860-1. Undetermined Language. PMID: 14915589. | No Full Text Available |
|  | 1. Sturdza N, Roman V, Nicolescu M, Alămiţă E, Borşai L. Human leptospirosis in Romania (1962-1966). Arch Roum Pathol Exp Microbiol. 1968 Mar;27(1):63-74. PMID: 5706452. | Research questions not answered |
|  | 1. Bal AM. Unusual clinical manifestations of leptospirosis. J Postgrad Med. 2005 Jul-Sep;51(3):179-83. PMID: 16333189. | Research questions not answered |
|  | 1. Panchabhai TS, Patil PD, Shah DR, Joshi AS. An autopsy study of maternal mortality: a tertiary healthcare perspective. J Postgrad Med. 2009 Jan-Mar;55(1):8-11. doi: 10.4103/0022-3859.48434. PMID: 19242071. | Research questions not answered |
|  | 1. Sullivan ND. Leptospirosis in animals and man. Aust Vet J. 1974 May;50(5):216-23. doi: 10.1111/j.1751-0813.1974.tb02367.x. PMID: 4414082. | Review |
|  | 1. McClure EM, Goldenberg RL. Infection and stillbirth. Semin Fetal Neonatal Med. 2009 Aug;14(4):182-9. doi: 10.1016/j.siny.2009.02.003. Epub 2009 Mar 12. PMID: 19285457; PMCID: PMC3962114. | Review |
|  | 1. Puliyath G, Singh S. Leptospirosis in pregnancy. Eur J Clin Microbiol Infect Dis. 2012 Oct;31(10):2491-6. doi: 10.1007/s10096-012-1625-7. Epub 2012 May 2. PMID: 22549729. | Review |
|  | Zoonotic infections in adults in the Bushbuckridge District of Mpumalanga Province. Quan, V.C. et al. International Journal of Infectious Diseases, Volume 21, 186 | Poster |
|  | 1. Thiermann AB, Frank RR. Human leptospirosis in Detroit and the role of rats as chronic carriers. Int J Zoonoses. 1980 Jun;7(1):62-72. PMID: 7461920. | Animal |
|  | | |
| Case Report/  Series | 1. Liverpool J, Francis S, Liverpool CE, Dean GT, Mendez DD. Leptospirosis: case reports of an outbreak in Guyana. Ann Trop Med Parasitol. 2008 Apr;102(3):239-45. doi: 10.1179/136485908X278784. PMID: 18348778. | No leptospirosis in pregnancy |
|  | 1. McIntosh, N. and T. Madhavan (1982). "Zoonoses at Henry Ford Hospital: clinical, epidemiologic, and therapeutic aspects." Henry Ford Hosp Med J 30(1): 7-10 | No leptospirosis in pregnancy |
|  | 1. Maszkiewicz, W., K. Ornat, B. Zarczynska and D. Irzynska (1979). "Spirochaetal septicemia in newborn infants." Polski tygodnik lekarski (Warsaw, Poland : 1960) 34(8): 301-302. | No leptospirosis in pregnancy |
|  | 1. Naidoo PM. Acute renal failure. Experience with 52 patients treated at Livingstone Hospital. S Afr Med J. 1976 Jan 17;50(3):78-80. PMID: 1251280. | No leptospirosis in pregnancy |
|  | Case Report Molecular characterization of *Leptospira sp* by multilocus variable number tandem repeat analysis (MLVA) from clinical samples: a case report 2015. [HélènePailhoriès](https://www.sciencedirect.com/science/article/pii/S1201971215001642#!) | No leptospirosis in pregnancy |
|  | 1. Sin, J. Hartill-Dadson, M. B Garvey. Z Hussain and H. Ghaffer (2013), “An unexpected diagnosis encountered in the hematology laboratory: Peripheral blood findings in a patient with relapsing fever.” International Journal of Laboratory Hematology 35 (SUPPL.1): 105 | No leptospirosis in pregnancy |
|  | 1. Guggenheim JN, Haverkamp AD. Tick-borne relapsing fever during pregnancy: a case report. J Reprod Med. 2005 Sep;50(9):727-9. PMID: 16363764. | No leptospirosis in pregnancy |
|  | 1. Davis RD, Burke JP, Wright LJ. Relapsing fever associated with ARDS in a parturient woman. A case report and review of the literature. Chest. 1992 Aug;102(2):630-2. doi: 10.1378/chest.102.2.630. PMID: 1643961. | No leptospirosis in pregnancy |
|  | 1. Samanta S, Samanta S, Haldar R. Emergency caesarean delivery in a patient with cerebral malaria-leptospira co infection: Anaesthetic and critical care considerations. *Indian J Anaesth*. 2014;58(1):55-58. doi:10.4103/0019-5049.126797 | Co-infection |
|  | 1. Yeap, Tat & Muniandy, Rajesh. (2019). CA-MRSA and Leptospirosis Co-Infections : The recipe to a Successful Management. Gazi Medical Journal. 30. 82-83. 10.12996/gmj.2019.23. | Co-infection |
|  | 1. Paranthakan, C., K. M. Ali, S. B. Sangeetha and S. M. Kumaravel (2018). "Fungal Pneumonia - Leptospirosis Co-Infection: A Case Report." Research Journal of Pharmaceutical Biological and Chemical Sciences 9(2): 251-253. | Co-infection |
|  | Chung HL, Ts'ao WC, Mo PS, Yen C. Transplacental or congenital infection of leptospirosis: clinical and experimental observations. Chin Med J (Engl) 1963;82:777-82. | No Full Text Available |
|  | | |
| Ongoing Studies | 1. Testing for leptospirosis infection in healthy donors. CTRI/2020/07/026640 | Research questions not answered |
|  | 1. [A study on utility of quick, fast and easy-to-do tests in improving care by decreasing the number of days of stay in hospital in patients who come with fever of short duration](https://apps.who.int/trialsearch/Trial2.aspx?TrialID=CTRI/2020/06/026174). CTRI/2020/06/026174 | Research questions not answered |
|  | 1. [To identify factors responsible for intensive care admission and death in patients suffering from acute febrile illness](https://apps.who.int/trialsearch/Trial2.aspx?TrialID=CTRI/2020/03/024355). CTRI/2020/03/024355 | Research questions not answered |
|  | 1. [Advancing Access to Diagnostic Innovation essential For AMR Prevention](https://apps.who.int/trialsearch/Trial2.aspx?TrialID=CTRI/2020/02/023253). CTRI/2020/02/023253 | Research questions not answered |
|  | 1. A non-randomized controlled clinical trial on the effectiveness of an education based intervention in improving the knowledge on leptospirosis that would help minimise delays in seeking treatment among farmers in the Monaragala district. SLCTR/2019/030 | Research questions not answered |
|  | 1. Comparison between dengue and leptospirosis. SLCTR/2019/030 | Research questions not answered |
|  | 1. Study of laboratory diagnosis of leptospirosis and its characterization in tertiary care hospital setting. CTRI/2019/01/017333 | Research questions not answered |
|  | 1. [Seroprevalence Study to Evaluate the Persistence of Anti-leptospira Antibody in Subjects Vaccinated With Spirolept Vaccine](https://apps.who.int/trialsearch/Trial2.aspx?TrialID=NCT03497572). NCT03497572 | Research questions not answered |
|  | 1. [The effectiveness and acceptability of the 'BALatrine': a culturally acceptable latrine intervention in resource limited environments.](https://apps.who.int/trialsearch/Trial2.aspx?TrialID=ACTRN12617000193370) ACTRN12617000193370 | Research questions not answered |
|  | 1. [Immune Response After Leptospirosis Infection](https://apps.who.int/trialsearch/Trial2.aspx?TrialID=NCT02898519). CT02898519 | Research questions not answered |
|  | 1. C[linical trial azithromycin versus doxycycline chemoprophylaxis in leptospirosis in farmers](https://apps.who.int/trialsearch/Trial2.aspx?TrialID=IRCT2015052322383N1). IRCT2015052322383N1 | Research questions not answered |
|  | 1. [Study of Decreasing Kinetics of the Leptospiremia During Antibiotic Treatment of Leptospirosis in Martinique](https://apps.who.int/trialsearch/Trial2.aspx?TrialID=NCT02000635). NCT02000635 | Research questions not answered |
|  | 1. [Proteomics study in patients with severe malaria](https://apps.who.int/trialsearch/Trial2.aspx?TrialID=CTRI/2013/08/003876). CTRI/2013/08/003876 | Research questions not answered |
|  | 1. [Rapid Diagnostic Tests and Clinical/Laboratory Predictors of Tropical Diseases In Patients With Persistent Fever in Cambodia, Nepal, Democratic Republic of the Congo and Sudan (NIDIAG-Fever)](https://apps.who.int/trialsearch/Trial2.aspx?TrialID=NCT01766830). NCT01766830 | Research questions not answered |
|  | 1. [French West Indies Leptospirosis Study](https://apps.who.int/trialsearch/Trial2.aspx?TrialID=NCT01607047). NCT01607047 | Research questions not answered |
|  | 1. [Usefulness of homoeopathic medicines in the management of Leptospirosis.](https://apps.who.int/trialsearch/Trial2.aspx?TrialID=CTRI/2008/091/000183) CTRI/2008/091/000183 | Pregnant people excluded |
|  | 1. [Use of corticosteroids in pulmonary leptospirosis: a randomised double-blind clinical trial](https://apps.who.int/trialsearch/Trial2.aspx?TrialID=ISRCTN74625030). ISRCTN74625030 | Pregnant people excluded |
|  | 1. [Desmopressin and Dexamethasone Adjunctive Treatment for Leptospirosis](https://apps.who.int/trialsearch/Trial2.aspx?TrialID=NCT00592566). NCT00592566 | Pregnant people excluded |
|  | 1. Homoeopathy as as add on treatment for severe Leptospirosis patients on conventional care. CTRI/2012/01/002316 | Pregnant people excluded |
|  | 1. Risk Factors and Chemoprophylaxis for Leptospirosis Infection during Urban Flooding in Southern Thailand: A Case-Control Study. TCTR20131106001 | Pregnant people excluded |
|  | 1. [‘Effectiveness and safety of the use of N-acetylcysteine in patients with kidney failure in leptospirosis’](https://apps.who.int/trialsearch/Trial2.aspx?TrialID=SLCTR/2018/016). SLCTR/2018/016 | Pregnant people excluded |
|  | 1. [Plasma exchange for lung haemorrhages in leptospirosis](https://apps.who.int/trialsearch/Trial2.aspx?TrialID=SLCTR/2018/013). SLCTR/2018/013 | Pregnant people excluded |
|  | 1. Comparing Two Antibiotic Therapy Periods (3 Versus 7 Days) in Patients With Mild Leptospirosis and Seen at the Hospital in 5 French Overseas Departments (Martinique, Guadeloupe, French Guiana, Reunion, Mayotte). NCT04211649 | Pregnant people excluded |
|  | 1. Risk factors for kidney damage in leptospirosis in infection. CTRI/2018/09/015849 | No further information available |
|  | 1. The effectiveness of N-acetylcystein (NAC) on accelerating renal failure recovery. IRCT20190727044343N4 | No further information available |
