# Supplementary material for: Leptospirosis in pregnancy: A systematic review
Source: PLoS Negl Trop Dis. 2021 Sep 14;15(9):e0009747. doi: 10.1371/journal.pntd.0009747 (PMC8462732; doi:10.1371/journal.pntd.0009747)
Supplement: Attachment — Submitted filename: Comments to the authors PNTD-D-21-00588.pdf [file pntd.0009747.s010.pdf]

Comments to the authors

This manuscript “Leptospirosis in Pregnancy: A systematic review by Selvarajah et al. is an excellent, well-written article. Given the limitation involved, the author has produced a useful overview that provides a very useful resource for current practitioners. The documentation and discussion appear descriptively appropriate. After careful consideration, the manuscript will likely to be suitable for publication if it is revised to address the minor points below.

Minor comments

Table 1, Study 3 [22], Quality: I think it should be changed “Good-9” to “moderate-9” according to the first sentence of page 18 (Line 183-185, The majority of observational studies had a moderate to high level of bias, being of ‘fair’ to ‘poor’ quality, except for one cross-sectional study (Study 1, Table 1 and 2) which was graded to be of ‘good’ quality.).

Edit MS for space and double full-stop (such as at line 84, 205, 434 and etc).

Check reference no. 3, line 491.

Typo

1. Abstract, introduction, 3rd sentence “was” should be “were”
2. Line 43; “underreported” to “underreporting”
3. Line 51; “to efficacy” to “ to the efficacy”
4. Line 96; “the sparsity of “ to “sparse”
5. Line 218; “Carribean” to “Caribbean”
6. Line 272; it is better to use the full name for the first time such as Microscopic Agglutination Test (MAT).
7. Line 417: “state” to “states”
