# Supplementary material for: Leptospirosis in pregnancy: A systematic review
Source: PLoS Negl Trop Dis. 2021 Sep 14;15(9):e0009747. doi: 10.1371/journal.pntd.0009747 (PMC8462732; doi:10.1371/journal.pntd.0009747)
Supplement: Attachment — Submitted filename: Letter of response.docx [file pntd.0009747.s011.docx]

The Editors

PLOS Neglected Tropical Diseases

28 July 2021

Re: Response to reviewers’ comments

Dear Editors,

Thank you for your time in considering our paper and coming back with overwhelmingly positive comments. We thank the reviewers for their constructive feedback. We have edited the manuscript based on the suggestions and have also submitted a point by point response to the reviewer comments. The edits on the revised manuscript are in track-changes and we have also submitted a clean copy. We have also uploaded revised versions of the Appendix to reflect an edit made, one with tracked changes and a clean version.

We will be happy to provide any other information or clarifications, if required.

Yours sincerely,

Dr Sujitha Selvarajah

**Response to reviewers’ comments**

Reviewer #1: Thank you for the invitation to review this ‘first’ systematic review of leptospirosis in pregnancy. The authors have made the best of limited data and produced a helpful review. I have only minor comments overall.

The ‘treat early’ recommendation needs to be tempered by risk benefit in relation to antimicrobial resistance. A low tolerance to treat in pregnancy results in overtreatment varying with the epidemiology. This is also supported by the findings in the section of global burden in pregnant women with symptoms: Pooling together two datasets, (Studies 1,3; Table 1 [20,22]) we calculated the incidence of leptospirosis in febrile pregnant patients to be 1.3 per 10,000 (95% CI 0.5 to 2.9 per 10,000). The calculated incidence of leptospirosis in pregnant patients with jaundice, pooling two datasets (Studies 2, 6; Table 1 - [21, 24]; Table 1) was comparable, 1.3 per 10,000 (95% CI 0.2 to 4.6 per 10,000). With such a low incidence in 'symptomatic' women overtreatment (without supportive diagnostics) is inevitable and not without consequence.

Response: We thank the reviewer for the constructive feedback and agree that a recommendation for treating early with antibiotics should be balanced with emerging antimicrobial resistance and in this context also discuss the importance of confirmatory diagnostics that are readily accessible. We have added the following in the ‘Discussion’ section of the revised manuscript: “We appreciate the benefits of treating a pregnant patient with suspected but not confirmed Leptospirosis with antibiotics must be weighed up with the risks of antimicrobial resistance, and this must be a risk assessment that each clinician considers.”

It would be helpful for the reader to have some context to the estimation of incidence. For PW the estimate is 1.3 per 10,000 in women presenting with fever or jaundice, which compared to an overall incidence estimate of 0.01 to 97.5 annual cases per 10,000 population [ref PLoS Negl Trop Dis. 2015 Sep; 9(9): e0003898. Published online 2015 Sep 17. oi: 10.1371/journal.pntd.0003898 Global Morbidity and Mortality of Leptospirosis: A Systematic Review Federico Costa,# 1 , 2 , 3 José E. Hagan,# 1 , 3 Juan Calcagno, 1 Michael Kane, 4 Paul Torgerson, 5 Martha S. Martinez-Silveira, 1 Claudia Stein, 6 Bernadette Abela-Ridder, 7 and Albert I. Ko 1 , 3 ,*

Response: As advised by the reviewer we have added further details to compare our calculated incidence rate with that provided in the suggested systematic review.

“We calculated the incidence of leptospirosis in pregnancy to be 1.3 per 10,000 in women presenting with fever or with jaundice, which falls in the lower end of the range of overall incidence of leptospirosis in the general population, estimated to be 0.01 to 97.5 annual cases per 10,000 population in another systematic review [[3]](https://paperpile.com/c/IpLTMc/929v). Incidence in the pregnant population could be higher in endemic areas.”

In Q 12 of the quality assessment tool “Were the outcome assessors blinded to the exposure status of participants?” How was this interpreted: ‘outcome’ of the acute febrile episode or ‘outcome’ of the pregnancy? McGready et al were blind to the outcome of the fever diagnosis as leptospirosis by MAT and Scrub typhus serology (and PCR) were done well after study completion (they were not available in the study area); while malaria was instant, dengue same day, and urine and blood culture results were known within a few days. Apart from fever and miscarriage or delivery on the same day as enrolment to the study (very rare), most cohort studies with an acute febrile episode and enrolment in pregnancy are blind to the birth outcome.

Response: We thank the reviewer for the clarification about the study by McGready et al and acknowledge that this was a difficult question to answer from the methodology reported in the papers. We defined exposure as ‘leptospirosis diagnosis’ and outcomes referred to maternal and fetal outcomes, as predefined in our research questions and protocol. The reviewer raised a valuable point about how exposure could be identified retrospectively and so we re-reviewed all eight observational studies to re-assess this quality criterion. When re-assessing we specifically looked for any explicit mention of outcome assessors being blinded to the exposure, or explicit mention of leptospirosis exposure being confirmed retrospectively. With this in mind, we still found ‘Study 1 by McGready et al. to score ‘N’, with no explicit mention of blinding of outcome assessors to leptospirosis exposure, nor explicit mention of leptospirosis diagnosis being made after the maternal and fetal outcomes had occurred. On reassessing all the papers, most of our assessments remained the same, except for ‘Study 4’ and ‘Study 8.’ These changes did not however affect our overall assessment of the quality of the studies. Study 4 explicitly stated that the exposure to leptospirosis was confirmed retrospectively as the serum samples were from a ‘previous study’ where the outcome of ‘spontaneous abortion’ had already been confirmed, so was changed from ‘N’ to ‘Y.’ Study 8 was changed from ‘NA’ to ‘N’ as patients’ outcome was known at start of the study (only those who had had a spontaneous abortion were included) and methods included patients filling in a questionnaire asking for potential exposure to zoonosis, prior to the serological confirmation of zoonotic disease, thus making blinding not possible. We only used information that was available in the published studies for the quality assessment process.

Doxycycline: consider rephrasing from ‘cannot be used in pregnancy’ in the discussion, to ‘remains an option in pregnancy’, or other less definitive phrase – see EXPERT OPINION ON DRUG SAFETY, 2016, VOL. 15, NO. 3, 367–382 <http://dx.doi.org/10.1517/14740338.2016.1133584>

Response: We have rephrased the use of doxycycline in the revised draft as: “Whilst Doxycycline is commonly avoided in pregnancy due to the presumed tetracycline effect on skeletal development and permanent teeth discolouration[[58]](https://paperpile.com/c/IpLTMc/ewTC)” and rephrased comment about use of tetracycline as:

“Finally, regarding treatment, despite tetracyclines being generally avoided in pregnancy, there is a long list of antibiotics that are suitable for use in pregnancy that can be effective against leptospirosis and should be used if suspected in accordance with local sensitivities.”

We should not discourage asking for risk factors in any febrile episode in pregnancy. There was no mention of conjunctival suffusion frequently a clue to help differentiate leptospirosis from malaria, dengue, scrub typhus and other rickettsial illness, ehrlichiosis and acute viral infections including influence(and now COVID-19). Maybe this could be mentioned as a deficiency of the studies? How many mention or report it? Clinical acumen was very low in McGready et al [after excluding malaria cases]: treat early would have resulted in 4 in 5 suspected women receiving antibiotics who don’t need them [clinical accuracy was 16.7% (1/5)].

Response:

We thank the reviewer for this comment. We found this to be a really valuable point and created a criteria for extracting this data from all included case reports. The new extracted information is summarised in the results section. We have also amended the Appendix to include this new criteria as follows:

“Conjunctival Suffusion:

1 = Conjunctival suffusion present or mention of red eyes

0 = No conjunctival suffusion stated/mention eye examination or some eye findings but no mention of conjunctival suffusion or state normal examination findings

NA = Cannot determine from information if there was conjunctival suffusion or eyes were examined”

As well as including these findings in Table 4 of the article, we added this in the results section:

When comparing differentials for fever, conjunctival suffusion, a sign that can sometimes be used to differentiate between Leptospirosis and other causes of febrile illnesses such as Malaria [[48]](https://paperpile.com/c/IpLTMc/39ch), could be helpful in the context of pregnancy, in helping distinguish between pregnancy related disorders such as PET and HELLP from Leptospirosis as a cause. Information on ocular examination was only available for 13 out of the 35 cases, and of these, 38% (n=5) of the women had conjunctival suffusion.

Add the units of the seroprevalence: seroprevalence of 18.6 +/- 3.6 overall? Assume it is %.

Response: We thank the reviewer for pointing out the omission. We have added % in the revised draft.

Consider suggesting a fever algorithm for diagnosis and treatment of pregnant women: Each place in the world that treats pregnant women with fever requires an algorithm (based on what they have available to them) to support optimal care [perhaps there are sufficient cohort studies that could collectively develop this – they would vary slightly based on local epidemiology]. It is not foolproof – in McGready et al there was a death in a treated patient and no cause was found by any of the diagnostics available. A fever algorithm embodies your other suggestions for: ‘ask’, ’good clinical exam’, ‘use lab diagnostics’…treat early. A diagnosis by exclusion can also be helpful to minimize overuse of antibiotics.

Azithromycin in McGready et al was used in the pregnancy fever algorithm because at the time it was thought a safe option for treatment of scrub typhus in the area but also covered leptospirosis which is less prevalent.

Response: We agree with the reviewer’s suggestion that there is a need for locally developed clinical algorithms to improve diagnosis and minimise the treatment with unnecessary antibiotics. We have accordingly added the following paragraph in the ‘Discussion’ section of the revised draft -

“We appreciate that the benefits of treating a patient with suspected but not confirmed leptospirosis with antibiotics must be weighed up with the risks of antimicrobial resistance — this must be a risk assessment that each clinician considers. A clinical algorithm in endemic areas for management and treatment of a febrile pregnant patient that is location-specific may be a useful aid to help clinicians with the decision-making process. A clinical algorithm also combines the opportunity to incorporate management of other local causes of febrile illness. Furthermore, this highlights the need for a rapid field-based test. The combination of our first recommendation of ‘asking early’ and assessing for likelihood of leptospirosis exposure, with a rapid field-based test that confirms diagnosis prior to commencing antibiotics, reduces the risk of antibiotic resistance without compromising patient safety through delayed or missed opportunities to diagnose and treat.”

The manuscript discussion could stress/highlight the inadequate laboratory diagnostics for confirmation of this widespread zoonosis and the very great need to for a field based tool. RDT for dengue and malaria are very useful in practice (both these diseases don't need antibiotics).

Response: We thank the reviewer for the comment and have further highlighted the importance of adequate laboratory diagnostics and need for field-based tests for early diagnosis and treatment of leptospirosis in pregnancy in the ‘Discussion section. The following has been added: “This also further highlights the need for an affordable rapid field based test. The combination of our first recommendation of ‘asking early’ and assessing for likelihood of leptospirosis exposure, with a rapid field based test that confirms diagnosis prior to commencing antibiotics, reduces the risk of antibiotic resistance without compromising patient safety through delayed or missed opportunities to diagnose and treat.”

Very minor

Reference 3 – it says website?

Missing punctuation: communicable diseases[1] needs a full stop

Check your formatting of references: sometimes before punctuation and other times after e.g after “over humanity. [2]” and before:” [6,10].”

Response: We thank the reviewer for the thorough and detailed review of the paper. We have re-checked and edited the revised draft to correct references, typos and punctuation errors.

Reviewer #2: Dear Editor,

This manuscript “Leptospirosis in Pregnancy: A systematic review by Selvarajah et al. is an excellent, well-written article. Given the complexity involved, the author has produced a number of positive and welcome outcomes, which offers a useful overview of current research and policy and the resulting bibliography which provides a very useful resource for current practitioners. It is one to which the author(s) have made significant contributions.

Please note that I am not familiar with the details of the Meta analysis so cannot comment on their appropriateness and accuracy.

I have no hesitation in recommending that it be accepted for publication after a few typos and other minor details have been attended to.

Best regards,

Vanaporn Wuthiekanun

Response: We thank the reviewer for acknowledging the importance of the systematic review. We have re-checked and edited the revised draft to correct typos and punctuation errors.

--------------------

<b>Summary and General Comments</b></br></br>

Use this section to provide overall comments, discuss strengths/weaknesses of the study, novelty, significance, general execution and scholarship. You may also include additional comments for the author, including concerns about dual publication, research ethics, or publication ethics. If requesting major revision, please articulate the new experiments that are needed.

Reviewer #1: not applicable

Reviewer #2: Comments to the authors

This manuscript “Leptospirosis in Pregnancy: A systematic review by Selvarajah et al. is an excellent, well-written article. Given the limitation involved, the author has produced a useful overview that provides a very useful resource for current practitioners. The documentation and discussion appear descriptively appropriate. After careful consideration, the manuscript will likely to be suitable for publication if it is revised to address the minor points below.

Minor comments

Response: We thank the reviewer for acknowledging the importance of the systematic review and for the constructive feedback.

Table 1, Study 3 [22], Quality: I think it should be changed “Good-9” to “moderate-9” according to the first sentence of page 18 (Line 183-185, The majority of observational studies had a moderate to high level of bias, being of ‘fair’ to ‘poor’ quality, except for one cross-sectional study (Study 1, Table 1 and 2) which was graded to be of ‘good’ quality.).

Edit MS for space and double full-stop (such as at line 84, 205, 434 and etc).

Response: We thank the reviewer for pointing out the transcribing errors. In Table 1, studies coded as ‘fair’ were incorrectly coded as ‘moderate’. We changed them all to ‘fair’ for consistency with Table 2 and the National Heart Lung Institute’s Quality Assessment Criteria. We use the term ‘fair’ instead of ‘moderate’ throughout the paper for consistency as this is the phrasing used by the National Heart Lung Institute when referring to their quality assessment tools.

Check reference no. 3, line 491.

Typo

1.	Abstract, introduction, 3rd sentence “was” should be “were”

2.	Line 43; “underreported” to “underreporting”

3.	Line 51; “to efficacy” to “ to the efficacy”

4.	Line 96; “the sparsity of “ to “sparse”

5.	Line 218; “Carribean” to “Caribbean”

6.	Line 272; it is better to use the full name for the first time such as Microscopic Agglutination Test (MAT).

7.	Line 417: “state” to “states”

Response: We have edited the draft and have corrected the typing errors pointed out by the reviewer. We did not use the term ‘Microscopic Agglutination Test’ here as we refer ‘agglutination tests’ as an umbrella term - including both some older case reports which refer to ‘agglutination tests/methods’ and case reports which specify ‘Microscopic Agglutination Tests.’
